# A Phylogenomic Census of Molecular Functions Identifies Modern Thermophilic Archaea as the Most Ancient Form of Cellular Life

**DOI:** 10.1155/2014/706468

**Published:** 2014-08-31

**Authors:** Arshan Nasir, Kyung Mo Kim, Gustavo Caetano-Anollés

**Affiliations:** ^1^Evolutionary Bioinformatics Laboratory, Department of Crop Sciences, and Illinois Informatics Institute, University of Illinois at Urbana-Champaign, Urbana, IL 61801, USA; ^2^Microbial Resource Center, Korea Research Institute of Bioscience and Biotechnology, Daejeon 305-806, Republic of Korea

## Abstract

The origins of diversified life remain mysterious despite considerable efforts devoted to untangling the roots of the universal tree of life. Here we reconstructed phylogenies that described the evolution of molecular functions and the evolution of species directly from a genomic census of gene ontology (GO) definitions. We sampled 249 free-living genomes spanning organisms in the three superkingdoms of life, Archaea, Bacteria, and Eukarya, and used the abundance of GO terms as molecular characters to produce rooted phylogenetic trees. Results revealed an early thermophilic origin of Archaea that was followed by genome reduction events in microbial superkingdoms. Eukaryal genomes displayed extraordinary functional diversity and were enriched with hundreds of novel molecular activities not detected in the akaryotic microbial cells. Remarkably, the majority of these novel functions appeared quite late in evolution, synchronized with the diversification of the eukaryal superkingdom. The distribution of GO terms in superkingdoms confirms that Archaea appears to be the simplest and most ancient form of cellular life, while Eukarya is the most diverse and recent.

## 1. Introduction

The tripartite nature of the cellular world is well established, with living organisms divided into three distinct life forms: Archaea, Bacteria, and Eukarya. Collectively, these groups are also referred to as the three “domains” or “superkingdoms” of life [1, 2]. Both Archaea and Bacteria are unicellular akaryotic microbes that generally lack a nucleus, mitochondria, and some membrane-bound organelles commonly found in the eukaryotic cells. Despite considerable morphological similarities between Archaea and Bacteria, they are recognized as distinct superkingdoms due to the presence of unique ribosomal proteins [3], differences in the composition and stereochemistry of cell wall lipids (glycerol-ether in Archaea versus glycerol-ester in Bacteria) [4, 5], and dissimilar DNA replication apparatus [6], habitats, and interactions with other cells [7]. Members of the archaeal superkingdom are generally found in extreme environments such as high temperatures and/or saline conditions (e.g.,* Methanopyrus kandleri* i.e., capable of surviving at 122°C [8]). In contrast, bacterial species are more widespread and are common pathogens. The superkingdom Eukarya includes a diverse group of both unicellular and multicellular organisms that contain many membrane-bound organelles (e.g., the nucleus) and complex cytoplasmic structures (e.g., cytoskeleton). In addition to the three generally accepted superkingdoms, recent studies also point to the existence of a fourth “supergroup” comprised of viruses with medium-to-large genomes (e.g., mimiviruses and megaviruses [9]). Thus giant viruses could be representatives of an ancient cellular mode of life that is distinct from extant cellular life forms [10].

Despite the fact that the three-domain classification of cellular life is widely accepted, the evolutionary relationships between the three superkingdoms remain largely unresolved. Initial phylogenetic studies based on ancient paralogous genes proposed a sister relationship between Archaea and Eukarya, both derived from a basal bacterial group. In fact this “canonical” rooting of the tree of life (ToL) has been recovered in a number of phylogenetic studies based on gene sequences (e.g., ATPases [11] and elongation factors [12]). The use of gene sequences in global phylogenetic analysis, however, has been challenged due to their inability to fully resolve the very deep basal relationships in the ToL (e.g., see [13] and references therein). In comparison, molecular structures are more conserved and robust than gene sequences [14]. For example, phylogenetic studies involving more conserved evolutionary characters such as structures of protein domains [15–19], tRNA [20, 21], 5S rRNA [22], RNase P [23], tRNA paralogs [24–27], and more recently molecular functions [28, 29] consistently identified Archaea as the most ancient superkingdom, while Bacteria and Eukarya formed derived sister groups.

**Figure 1:**
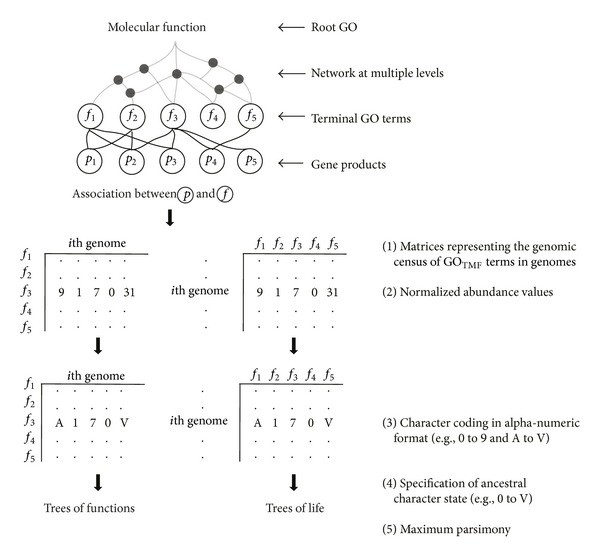
Overview of the phylogenomic methodology. A matrix of raw census of GO_TMF_ terms was normalized, standardized, and rescaled for phylogenetic reconstruction. Trees of functions (ToFs) were polarized by maximum character state (i.e., V) while trees of life were polarized (ToLs) by the minimum value (0) in the matrix.

Here, we revisit the evolutionary relationships between the three superkingdoms by reconstructing phylogenies using a novel and relatively understudied set of phylogenetic characters. We utilized the functional information of gene products defined by the gene ontology (GO) database [30, 31] as molecular characters to distinguish cellular species that have been fully sequenced. The GO is structured into three main hierarchies: (i)* molecular function*, (ii)* biological process*, and (iii)* cellular component*. Each of these hierarchies descends into multiple levels in the form of directed acyclic graphs (DAGs), where child GO terms can be associated with multiple parent terms and vice versa. The GO terms at higher hierarchical levels generally represent more broad functions (e.g., “catalytic activity [GO: 0003824]”), while terms at lower and terminal levels represent more specialized activities (“ferredoxin-NADP+ reductase activity [GO: 0004324]”). Thus, GO hierarchies are consistent with the generally accepted assumptions that ancient molecules were multifunctional with broad specificity (i.e., comparable to higher level GO terms), while modern molecules represent highly specialized functions (terminal GO terms) [32–34]. Therefore, the structure of GO hierarchies and their intimate association with organism physiology makes them strong candidates for use in phylogenetic studies [35]. GO terms are however structured as DAGs and are therefore prone to convergent evolution as one child GO term can have multiple parents. This factor could complicate phylogenetic inferences when GO terms are used as phylogenetic characters. To minimize the effects of such non-vertical evolutionary processes structured by the DAGs, we sampled only the terminal GO terms of the* molecular function* hierarchy (hereinafter GO_TMF_ terms) (Figure 1), as they represent the most specialized molecular activities of the cell and provide integrative views about organism physiology. To further protect from horizontal gene transfer (HGT) that is believed to occur in microbial species with high frequency [36], we excluded GO_TMF_ terms from the analysis that were identified as probable subjects for HGT. The analysis yielded phylogenies and distributions that described novel patterns in the evolution of cells and were compatible with traditional classifications, thereby supporting the choice of GO_TMF_ terms as molecular characters in evolutionary studies. Results revealed an early thermophilic origin of the archaeal superkingdom, global trends of genome reduction in microbial superkingdoms, and significant expansion of eukaryal diversity late in evolution.

## 2. Materials and Methods

### 2.1. Data Retrieval and Manipulation

Recently, we used GO terms in an ahistorical (i.e., non-evolutionary) and phylogenomic exercise to report the evolution of modern cells [28, 29]. Here, we reused the dataset to confirm the inferences drawn in [28, 29] by conducting specific analyses on the origin and spread of GO terms in modern cells. Details about data extraction and manipulation can be found in [28, 29]. Specifically, we downloaded the Gene Ontology Association (GOA) files for a total of 1,595 organisms from the European Bioinformatics Institute website (http://www.ebi.ac.uk/GOA/proteomes, November 2009). We filtered organisms that were redundantly present, excluded multiple strains of the same bacterial species keeping only the type strains, and used a threshold of 50% coverage (i.e., number of proteins assigned to GO_TMF_ terms divided by the total number of proteins) to exclude low quality genomes from the analysis. We also excluded GOA files for organisms exhibiting either facultative parasitic or parasitic lifestyles as they coevolve with their hosts and introduce biases into the global phylogenomic analyses [17, 18]. Organism lifestyles were studied using various online resources such as the genomes online database (GOLD) [37, 38] and previously published data [39]. This reduced the dataset to 249 free-living organisms including 45 Archaea, 183 Bacteria, and 21 Eukarya (See Table S1 in Supplementary Material is available online at http://dx.doi.org/10.1155/2014/706468). A total of 2,039 nonredundant GO_TMF_ terms were detected in the genomes of these organisms.

### 2.2. Exclusion of HGT-Acquired GO_TMF_ Terms

We identified 72 archaeal and bacterial genomes from our dataset that were cross-listed in the horizontal gene transfer database (HGT-DB) [40]. Protein sequences from these genomes were retrieved from the HGT-DB and compared with the corresponding GOA files. Specifically, we extracted the GO annotations for horizontally transferred proteins (HTPs) for genomes common in our GOA files and in HGT-DB. This set of GO_TMF_ terms was likely acquired by HGT.

For confirmation purposes, we performed a statistical hypergeometric distribution test to determine what HGT-acquired GO_TMF_ terms were significantly enriched in our dataset (see [35, 41] for details). This resulted in the identification of 115 GO_TMF_ terms that were potential candidates for HGT. The exclusion of HGT-acquired GO_TMF_ terms resulted in the final dataset of 249 free-living genomes and a repertoire of 1,924 GO_TMF_ terms. We note that resulting dataset was likely minimally affected by HGT (and other non-vertical evolutionary processes) because both the parasitic organisms and the GO_TMF_ terms most likely acquired via HGT were excluded from the analysis. Moreover, phylogenetic and network studies confirmed that once the HGT-derived characters were excluded, the resulting phylogenies performed in a way superior to traditional sequence-based trees and had minimal conflict [29]. The dataset however retains the evolutionarily deep proteome-shaping effects of endosymbiotic events that likely tailored the eukaryotic cell.

### 2.3. Phylogenomic Analysis

We used previously described methodology to reconstruct trees of functions (ToFs) and ToLs portraying, respectively, the evolution of GO_TMF_ terms and species [19, 29, 35, 42]. We first counted the number of times each GO_TMF_ term was present in every genome and generated a matrix representing the census of molecular functions in genomes (Figure 1). The raw counts of the genomic abundance of each GO_TMF_ term in every genome (*g*_ab_) were log-transformed to account for unequal genome sizes and heterogeneous variances and then divided by the maximum abundance value (*g*_max⁡_) in the matrix. The standardized counts were then rescaled from 0 to 31 using an alphanumeric format (0–9 and A–V) to allow compatibility with PAUP phylogenetic reconstruction software (ver. 4.0b10) (Figure 1) [43]. The equation below describes the data manipulation procedure [16, 19]:

(1) $$\begin{array}{c} g_{\text{ab}\_\mathrm{norm}}=\text{Round}\left[\frac{\ln\left(g_{\text{ab}}+1\right)}{\ln\left(g_{\max}+1\right)}\right]*31. \end{array}$$

Maximum parsimony (MP) was used to search for the most parsimonious tree describing the evolution of ToFs and ToLs with minimum possible character changes. We note that MP performs superior to maximum likelihood when dealing with multistate phylogenetic characters evolving under different evolutionary rates [44] (e.g., GO_TMF_ terms that are accumulated in genomes at different evolutionary rates). Moreover, normalization and rescaling of raw abundance values into 32 possible character states ensure compatibility with PAUP∗ and reduce the likelihood of convergent evolution. Phylogenetic trees were intrinsically rooted using the Lundberg method that places the root at the most parsimonious location without any outgroup taxa specification [45].

For the ToFs, we assumed that the most abundant molecular function appeared first in evolution (i.e., we rooted trees by maximum value in the matrix by specifying V as the ancestral character state) [35]. In contrast, ToLs were rooted by the smallest value in the matrix (i.e., character state 0) under the assumption that the ancestral genome had very limited functional capabilities and it progressively enhanced its repertoire of molecular functions [17, 18, 29]. The reliability of phylogenetic trees was evaluated by 1,000 bootstraps. Trees were visualized using Dendroscope ver. 3.2.7 [46].

### 2.4. Estimating the Origin of GO_TMF_ Terms

From the ToF, we calculated the distance of each taxon (i.e., GO_TMF_ term) from the root by counting the number of nodes from a given position to the base and dividing by the total number of taxa. This node distance (*nd*) was used to estimate the relative age of each GO_TMF_ term on a scale from 0 (most ancient) to 1 (most recent). The* nd* has been successfully utilized previously in the evolutionary study of protein domain structure [15] and closely follows a molecular clock [47]. Thus,* nd* can be reliably used as a proxy to infer evolutionary time and genomic appearance of molecular functions.

### 2.5. Popularity of GO_TMF_ Terms in Genomes

To study the spread of GO_TMF_ terms in genomes, we used a distribution index (*f* value) to quantify the popularity of molecular functions. This index was computed by the number of genomes encoding a particular GO_TMF_ term divided by the total number of genomes, on a scale from 0 to 1. Thus, an *f* value of 0 indicates complete absence of a GO_TMF_ term whereas a value close to 1 indicates near universal presence. Molecular activities that are vital to cellular life were expected to have higher *f* values, while GO_TMF_ terms unique to a species or superkingdom were anticipated to have lower *f* values.

### 2.6. Persistence Strategies of Organisms

We used previously described concepts of economy, flexibility, and robustness to determine the persistence strategies of organisms in our dataset [48]. Economy was defined by the total number of nonredundant GO_TMF_ terms present in a genome. Thus genomes with low economy harbor limited molecular activities and persist with a parsimonious strategy. Flexibility was defined by the total (i.e., redundant) number of GO_TMF_ terms in a genome. Thus, genomes with high flexibility encode multiple instances of the same GO_TMF_ term and therefore confer flexibility to the organismal make up. Finally, robustness was defined by the ratio of flexibility to economy, indicating increased resistance to environmental stress and the ability to survive damage. In other words, flexibility is the ability of an organism to respond similarly to different levels of the same stimuli (e.g., various intensities of light) whereas robustness is the ability to withstand a diverse array of stimuli without innovating new modules [48].

## 3. Results and Discussion

### 3.1. Functional Diversity in Superkingdoms

**Figure 2:**
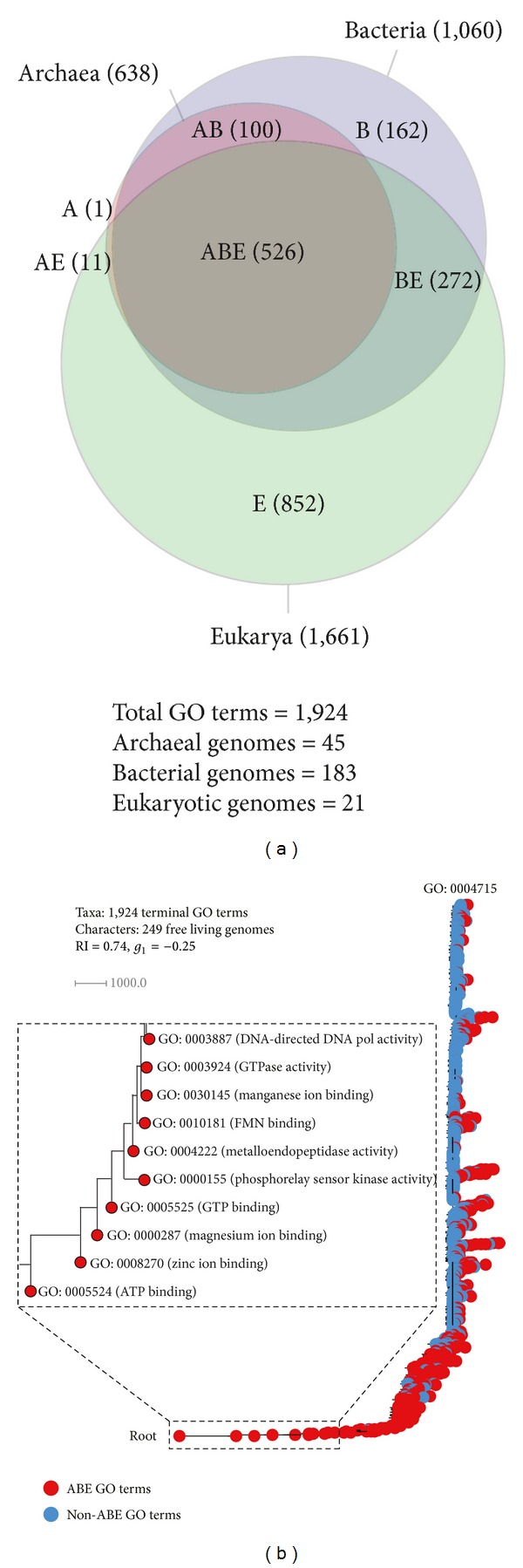
The distribution and evolution of GO_TMF_ terms. (a) A Venn diagram illustrates the sharing patterns of molecular functions in the seven taxonomic groups (reproduced from [28]). Numbers of terms in Venn taxonomic groups and in superkingdoms are given in parentheses and are reflected by the areas of the diagram. (b) A ToF (tree length = 99,594 steps) portraying the evolution of GO_TMF_ terms. Molecular activities present in all three superkingdoms are colored red while those unique to a superkingdom or shared by at most two are colored blue. The inset displays the most basal taxa. GO: 0004715 is the “nonmembrane spanning protein tyrosine kinase activity.”

A Venn diagram revealed the distribution patterns of 1,924 GO_TMF_ terms in the three superkingdoms (Figure 2(a)) (reproduced from [28]). These included GO_TMF_ terms that were uniquely present in a superkingdom (i.e., A, B, and E), were shared by two superkingdoms (AB, AE, and BE), or were universal (ABE), thus resulting in seven possible Venn taxonomic groups made explicit in Figure 2(a). Nearly 44% of the total GO_TMF_ terms were uniquely detected in Eukarya (E), demonstrating the massive functional diversity of eukaryal organisms. In contrast, only 8.4% and 0.05% GO_TMF_ terms were exclusive to Bacteria (B) and Archaea (A), respectively (Figure 2(a)).

The massive number of unique eukaryal molecular functions is a significant outcome considering we sampled only 21 eukaryal genomes compared to 45 and 183 genomes from organisms in Archaea and Bacteria, respectively. The result indicates that Eukarya likely discovered a large number of novel molecular activities throughout the course of evolution. Previous analyses suggested that gene duplications and rearrangements were abundant during the evolution of eukaryal superkingdom and played an important role in tailoring the eukaryotic genomes [16, 49]. We propose that increased rates of these events led to the rapid functional diversification of ancient promiscuous molecules into molecules with more advanced and novel functional capabilities, thereby increasing the functional repertoire of eukaryotic cells. In contrast, akaryotes persisted with a strategy of economy and harbored simpler functional profiles.

A total of 526 GO_TMF_ terms were present in all three superkingdoms and made the second-largest Venn taxonomic group (ABE) (Figure 2(a)). The number of GO_TMF_ terms shared between any two superkingdoms was highest for the BE group (272), intermediate for AB (100), and lowest for AE (11) (Figure 2(a)). One explanation for the very large size of the BE taxonomic group is bacterial endosymbiosis during the evolution of eukaryotes that likely transferred many bacterial genes to the host cell [50]. However, we filtered parasitic organisms from our dataset and this also resulted in the exclusion of genus* Rickettsia* (obligate intracellular parasites) that is believed by some to be the ancestor of modern mitochondria [51]. Moreover, bacterial proteins that were likely subjects of HGT were also eliminated by the statistical enrichment test (see Section 2). Thus, our data is more compatible with an alternative scenario in which both Bacteria and Eukarya evolved from a complex and rich ancestor of extant life while Archaea evolved first by massive genome streamlining. In other words, the very large size of the BE taxonomic group (i.e., 272 GO_TMF_ terms) cannot solely be explained by endosymbiosis and likely represents a strong vertical trace from the rich community of ancestral cells (anticipated in [28]).

To conclude, it is evident from the Venn diagram that Archaea represents the simplest form of cellular life. Archaeal proteomes are functionally least diverse and thrive with a minimal repertoire of molecular activities. Bacteria follow an intermediate route that is more like Archaea than Eukarya, while the latter is functionally rich and encodes richer genomes.

### 3.2. Evolution of Molecular Functions

A ToF described the evolution of 1,924 GO_TMF_ terms (taxa) in 249 free-living organisms (characters) (Figure 2(b)). The ten most basal taxa corresponded to important catalytic and binding activities, including “ATP binding [GO: 0005524],” “zinc ion binding [GO: 0008270],” “magnesium ion binding [GO: 0000287],” “GTP binding [GO: 0005525],” “phosphorelay sensor kinase activity [GO: 0000155],” “metalloendopeptidase activity [GO: 0004222],” “FMN binding [GO: 0010181],” “manganese ion binding [GO: 0030145],” “GTPase activity [GO: 0003924],” and “DNA-directed DNA polymerase activity [GO: 0003887]” (inset in Figure 2(b)). “ATP binding” was the most ancient molecular function while “nonmembrane spanning protein kinase activity [GO: 0004715]” the most derived (Figure 2(b)). The majority of the universal GO_TMF_ terms occupied deep positions in the phylogeny (red circles), while molecular activities unique to or shared by at most two superkingdoms (i.e., AB, AE, and BE taxonomic groups) appeared late (blue circles) and were derived from the ancient molecular functions (Figure 2(b)). The very early appearance of metabolic functions matches results from previous evolutionary studies of protein domain structure and molecular functions (e.g., [15–17, 35]).

**Figure 3:**
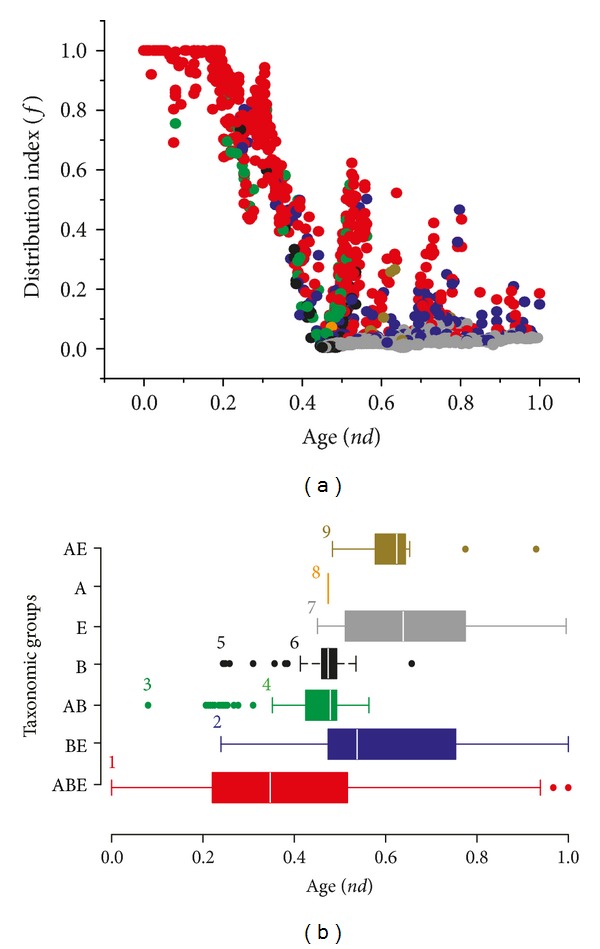
Order of the evolutionary appearance of Venn taxonomic groups. (a) Scatter plot highlighting the distribution of GO_TMF_ terms with respect to evolutionary time (*nd*) and distribution in genomes (*f*). (b) Boxplots displaying the distribution of GO_TMF_ terms with respect to evolutionary time (*nd*) in the seven taxonomic groups. The most ancient GO_TMF_ term in each taxonomic group (and outliers) is indexed with numbers 1, “ATP binding [GO: 0005524]”; 2, “DNA replication origin binding [GO: 0003688]”; 3, “penicillin binding [GO: 0008658]”; 4, “2,3,4,5-tetrahydropyridine-2,6-dicarboxylate N-succinyltransferase activity [GO: 0008666]”; 5, “UDP-N-acetylmuramoylalanyl-D-glutamyl-2,6-diaminopimelate-D-alanyl-D-alanine ligase activity [GO: 0008766]”; 6, “chorismate lyase activity [GO: 0008813]”; 7, “CCR1 chemokine receptor binding [GO: 0031726]”; 8, “methylenetetrahydromethanopterin dehydrogenase activity [GO: 0030268]”; and 9, “nicotinamine synthase activity [GO: 0030410]”.

To unfold the order of appearance of molecular functions in evolutionary time, we calculated a node distance (*nd*) representing the relative age of each GO_TMF_ term directly from the ToF (see Materials and Methods). We plotted the* nd* values of GO_TMF_ terms against a distribution index (*f*), defined by the total number of genomes encoding a GO_TMF_ term divided by the total number of genomes, to study the popularity and distribution of GO_TMF_ terms in superkingdom groups (Figure 3). The* nd* versus *f* plot revealed remarkable and unprecedented evolutionary patterns (Figure 3(a)).

The majority of the most ancient molecular functions (0 ≤ *nd* ≤ 0.2) were universally present (red circles) with remarkably high *f* values (Figure 3(a)). In fact, a total of 26 GO_TMF_ terms had an *f* equal to 1 indicating ubiquitous presence in all the genomes that were sampled (Table 1). These universal GO_TMF_ terms corresponded to fundamental catalytic and binding activities that are crucial for life such as binding to ATP [GO: 0005524], DNA replication [GO: 0003887], cleavage of RNA-DNA hybrids [GO: 0004523], unwinding of DNA strand before replication and transcription [GO: 0003917], biosynthetic activities of aminoacyl-tRNA synthetases [GO: 0004813, GO: 0004815, GO: 0004820, GO: 0004821, GO: 0004824, GO: 0004826, GO: 0004831], and others listed in Table 1. Remarkably, all these universal GO_TMF_ terms appeared very early in evolution (*nd* < 0.2) (Table 1) and before the appearance of superkingdom-specific GO terms (read below). The list indicates the last universal common ancestor had a cell-like make up with complex catalytic machinery already present, as suggested by previous studies of protein domains and molecular functions [17, 18, 35].

However,* f* started to drop with the progression of* nd* and approached 0 at* nd* 0.45. We observed that a considerable fraction of BE (blue circles) and AB (green) GO_TMF_ terms appeared before *nd* = 0.45, suggesting that reductive evolutionary processes were at play (Figure 3(a)). We propose that very early in evolution the probability of one lineage completely loosing a GO_TMF_ term was greater than the probability of the other two lineages acquiring the same GO_TMF_ term concurrently. Thus, appearances of BE and AB taxonomic groups most likely represented complete loss events of GO_TMF_ terms in Archaea and Eukarya (resp.,) that started to occur very early in evolution (read below). In contrast, B (black), E (grey), A (orange), and AE (antique bronze) GO_TMF_ terms appeared predominantly during the late evolutionary period (*nd* > 0.45). Eukaryotes, in particular, discovered a massive number of novel GO_TMF_ terms late in evolution, thereby compensating for the early reductive events.

The boxplots in Figure 3(b) confirmed the appearance order of taxonomic groups in evolution. The first molecular activity to appear in evolution was “ATP binding [GO: 0005524]” at *nd* = 0 (Figure 3). The ABE taxonomic group ranged from *nd* = 0 to *nd* = 1 and was followed by the appearances of BE, AB, B, E, A, and AE, in that order (Figure 3(b)). Although, few members of the AB taxonomic group appeared earlier than BE, they were identified as outliers and were likely candidates of HGT that occurred between Archaea and Bacteria later on in evolution (e.g., “penicillin binding [GO: 0008658]”) (Table 2 for outliers, also read below). Thus, the BE group probably appeared before the AB group signaling the first complete loss event of a GO_TMF_ term in any superkingdom. This intuition is also in line with previously published analyses that also proposed evolution of Archaea by primordial genome reduction events (e.g., [15, 52]). Our results therefore support the early split of Archaea from an evolving world of primordial organisms by following a path to genome reduction that ultimately led to the poor representation of GO_TMF_ terms in the archaeal taxonomic groups (i.e., A, AE, and AB) of the Venn diagram (Figure 2(a)).

The first molecular functions unique to the BE group were “DNA replication origin binding [GO: 0003688],” “[acyl-carrier-protein] S-malonyltransferase activity [GO: 0004314],” and “FMN adenyltransferase activity [GO: 0003919].” These three GO_TMF_ terms appeared jointly at *nd* = 0.24 (boxplot for BE in Figure 3(b)). As stated above, this event also represents the first complete loss event of a GO_TMF_ term in Archaea. It is interesting to note that none of the archaeal proteins were annotated to the GO: 0003688 GO_TMF_ term in our dataset. Interestingly, archaeal genomes lack homologues of replication proteins that play important roles in regulating the initiation of DNA replication (e.g., Hda, YabA, Dam, or SeqA) [53]. An alternative explanation, though less likely, is the use of relatively low quality GO data for archaeal genomes. Archaea are understudied compared to Bacteria and Eukarya and this could reflect in missing crucial GO annotations for archaeal organisms. However, we discovered that GO coverage did not vary significantly among superkingdoms. For example, mean GO coverage in Archaea was 57%, which was not so far away from 60% coverage in both Bacteria and Eukarya (Table S1). Thus, complete absence of GO: 0003688 in Archaea is biologically significant and merits future work in the identification of archaeal homologs of bacterial and eukaryal proteins.

GO_TMF_ terms unique to superkingdoms started to appear late in evolution (*nd* > 0.4), first in Bacteria at *nd* = 0.41 (“chorismate lyase activity [GO: 0008813]”), then in Eukarya at *nd* = 0.45 (“CCR1 chemokine receptor binding [GO: 0031726]”), and finally in Archaea at *nd* = 0.47 (“methylenetetrahydromethanopterin dehydrogenase activity [GO: 0030268]”) (Figure 3(b)). “chorismate lyase activity” is important for the removal of pyruvate from chorismate and was first studied in* Escherichia coli* and other Gram-negative bacteria [54, 55]. “CCR1 chemokine receptor binding” activity is important during inflammatory responses to injuries and pathogens [56] and appeared uniquely in Eukarya at *nd* = 0.45. Finally, the archaeal-specific GO term (“methylenetetrahydromethanopterin dehydrogenase activity”) is involved in folic acid biosynthesis and was first studied in the hyperthermophilic archaeal species* Methanobacterium thermoautotrophicum* [57]. We also note that the AE taxonomic group appeared soon after the appearances of the A and E groups at *nd* = 0.48 (“nicotianamine synthase activity [GO: 0030410]”) indicating that Archaea and Eukarya were more similar to each other with respect to “modern” molecular activities (*nd* > 0.47) relative to the more ancient ones [58].

### 3.3. Global Tendencies in Superkingdoms

**Figure 4:**
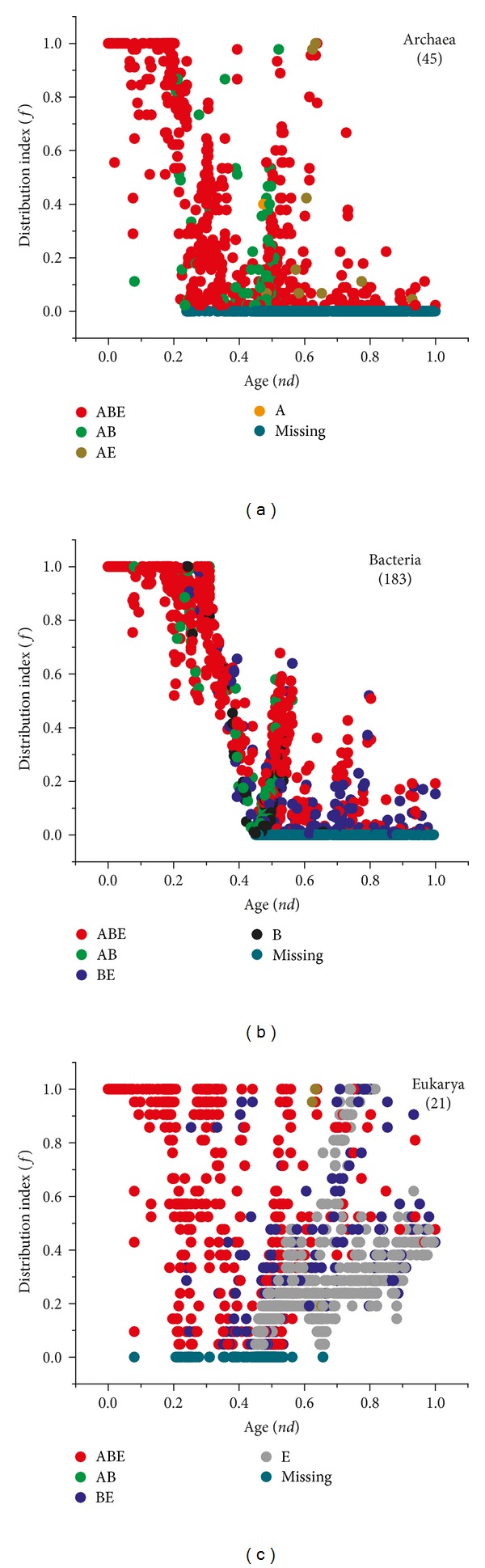
Scatter plots displaying the distribution of GO_TMF_ terms with respect to evolutionary time (*nd*) in Archaea (a), Bacteria (b), and Eukarya (c).

The *nd* versus *f* plots for individual superkingdoms confirmed earlier patterns (Figure 4). A total of 55 GO_TMF_ terms had an *f* of 1 indicating their ubiquitous presence within Archaea (Figure 4(a)). However, *f* started to drop rapidly with an increase in *nd*. The first complete loss event was recorded at *nd* = 0.23 for three GO_TMF_ terms “DNA replication origin binding,” “[acyl-carrier-protein] S-malonyltransferase activity,” and “FMN adenyltransferase activity” (cyan circles in Figure 4(a)). The GO_TMF_ terms unique to A and AE appeared later in evolution (*nd* > 0.45) and were distributed with low *f* values (Figure 4(a)).

In Bacteria, 56 GO_TMF_ terms were universally present in the bacterial genomes (*f* = 1) and had ancient origins (*nd* < 0.31). The *f* value started to drop and reached 0 at *nd* = 0.45 when the first complete loss event for “CCR1 chemokine receptor binding” was recorded. Alternatively, this molecular activity was likely never gained by the bacterial genomes and appeared uniquely in Eukarya conferring immunological capabilities to eukaryotic cells. The distribution of molecular functions in Bacteria was remarkably similar to the global distribution (Figure 3(a)), where most of the ancient GO_TMF_ terms were distributed with significantly higher *f* values, while the more derived ones distributed with smaller values (Figure 4(b)).

Finally, Eukarya exhibited remarkable variability in the spread of GO_TMF_ terms. A total of 125 GO_TMF_ terms were universally present in the eukaryotic genomes spanning the *nd* range from 0 to 0.8 (Figure 4(c)). The first complete loss event occurred at *nd* = 0.08 when “penicillin binding” activity was lost from the eukaryotic genomes. However, as explained previously, this GO_TMF_ term was an outlier in the AB taxonomic group (boxplot for AB in Figure 3(b)) and most likely represented a lateral acquisition event that occurred between akaryotic microbes. In fact, the term was universal in Bacteria (*f* = 1.0) but rare in Archaea (detected in only ~10% archaeal species) (Figures 4(a) and 4(b)). This suggested a late gene transfer from Bacteria to Archaea, once bacterial species appeared in the evolutionary scene. Similar patterns of transfer were also evident in other GO_TMF_ terms of ancient origin (*nd* < 0.3) of the AB group. Remarkably, the average *f* of these ancient GO_TMF_ terms was 0.31 in Archaea and 0.83 in Bacteria. This suggested that ancient GO_TMF_ terms were laterally transferred from Bacteria to Archaea, and not vice versa. We observed that the overall average *f* for all AB GO_TMF_ terms was 0.16 in Archaea and 0.26 in Bacteria (overall medians were 0.06 and 0.12, resp.). The GO_TMF_ terms of the AB taxonomical group listed in Table 2 are therefore atypical and likely represent ancient episodes of lateral transfer that merit further attention. Given this atypical behavior, the actual loss of a molecular activity in Eukarya occurred much later and after the first loss event in Archaea (Figure 3(b)).

Molecular functions unique to eukaryotes appeared (grey circles) late (*nd* < 0.45), just like those of Archaea and exhibited a tendency to become widespread in the eukaryotic species (Figure 4(c)). The exercise revealed that Archaea persisted with a parsimonious strategy while both Bacteria and Eukarya enriched their functional toolkits. In particular, eukaryotes acquired a large number of novel molecular activities very late in evolution suggesting a late diversification of the eukaryal superkingdom and explaining the remarkable diversity of species and levels of organization we observe today in Eukarya.

### 3.4. Evolution of Species

**Figure 5:**
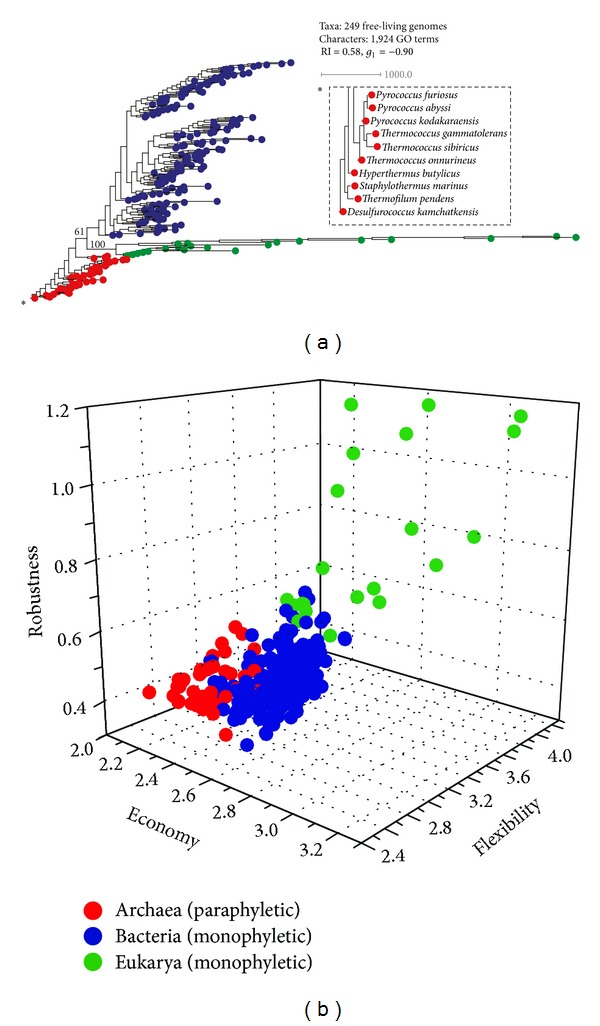
The tripartite division of the cellular world. (a) A ToL (tree length = 87,892) generated from the genomic census of GO_TMF_ terms in 249 free-living genomes resolves the three primary superkingdoms. Archaeal species (red) occupy the most basal positions in a paraphyletic manner, while monophyletic Bacteria (blue) and Eukarya (green) are evolutionarily derived. Numbers on branches indicate bootstrap support values. (b) A 3D-scatter plot dissects organisms into three superkingdoms: Archaea, Bacteria, and Eukarya. Genomes are labeled as in (a).

We reconstructed a ToL that described the evolution of 249 free-living organisms (taxa) using the repertoire of 1,924 GO_TMF_ terms as phylogenetic characters (Figure 5(a)). In agreement with the results obtained from ToFs, the ToL obtained from the genomic census of molecular functions suggested an ancient origin of Archaea and the late appearances of Bacteria and Eukarya (Figure 5(a)). Archaeal species occupied the most basal positions in a paraphyletic manner while both Bacteria and Eukarya formed monophyletic groups. The monophyletic eukaryal clade was highly supported (100% bootstrap). The most basal ToL taxa corresponded to thermophilic and hyperthermophilic archaeal species (e.g.,* Desulfurococcus kamchatkensis*,* Thermofilum pendens*,* Staphylothermus marinus*,* Hyperthermus butylicus*, along with species belonging to genera* Thermococcus* and* Pyrococcus*) suggesting a thermophilic origin of cellular life (inset in Figure 5(a)). However, the paraphyletic rooting of the ToL in Archaea demands an explanation. Our data showed that paraphyly was a consequence of an uneven distribution of GO_TMF_ terms in archaeal genomes. The differential patterns of loss of molecular functions in archaeal organisms were evident in Figure 4(a). This suggested that the last common archaeal ancestor was likely more complex than any of the extant archaeal genomes [59]. Perhaps, streamlining the molecular repertoire was better suited for nascent archaeal lineages when adapting to harsh environments on Earth. This “divergence-by-isolation” scenario could result in a paraphyletic snapshot of archaeal history in modern tree reconstructions.

We discovered that major archaeal groups, Crenarchaeota and Euryarchaeota, did not form cohesive groups. This is in conflict with a previously published phylogeny of archaeal species based on concatenated ribosomal proteins [60]. As explained above, non-cohesiveness of major archaeal phyla could be explained by the patchy distribution of GO_TMF_ terms in archaeal genomes. An alternative explanation is the limited sampling of archaeal species in our study. At the time of the analysis (Nov 2009), only 45 free-living archaeal organisms (mostly extremophiles) with >50% GO coverage were available for evolutionary study. Since then, our knowledge about Archaea has expanded considerably with the discovery of many mesophilic archaeal species. It will be important to include more archaeal genomes in future studies for verification purposes. Therefore we caution that patterns reported in the study are the most likely scenarios drawn from our data but crucially present a “functional” perspective to the evolution of modern cells that is different from the gene-centric sequence-based perspectives.

To further confirm the early origins of the archaeal superkingdom and inspired by a model of persistence strategies for cellular diversification [48], we calculated metrics for economy (total number of unique GO_TMF_ terms in a genome), flexibility (total redundant number of GO_TMF_ terms in a genome), and robustness (ratio of flexibility to economy) for all genomes in our dataset. These metrics describe strategies of deployment of molecular functions necessary for persistence as organisms sense and adapt to the environment. When plotted together in a 3D-scatter plot, archaeal genomes (red circles) occupied positions in close proximity to the origin indicating greatest economy but least flexibility and robustness (Figure 5(b)). In contrast, bacterial genomes exhibited intermediate levels of economy, flexibility, and robustness and were tightly clustered. This indicated that functional constraints on bacterial species were remarkably conserved. The eukaryal genomes displayed the lowest levels of economy but the greatest levels of flexibility and robustness and were distributed with greatest variability (Figure 5(b)). The exercise further strengthened the hypothesis that eukaryotes persist by fostering functional complexity while akaryotic microbes foster economy. Moreover, Archaea represent the simplest form of cellular life and appeared first in evolution.

### 3.5. Comparison with Competing Hypotheses

Our experiments predicted a thermophilic origin of diversified life (also anticipated in [61–63]) and challenged theories attributing the origin of life in colder environments (e.g., [64]). The results also did not support the origin of eukaryotes by fusion or interaction of two akaryotic cells [50, 65, 66]. Instead, our data show that the primordial stem line was enriched in molecular activities and gave birth first to Archaea, then Bacteria, and finally Eukarya (Figure 3). Eukaryal genomes likely retained many of the ancient molecular activities that were progressively lost from akaryotic microbes. Akaryotes compensated this loss by adapting to harsh environments and enjoying rapid growth cycles, possibly under pressure from cellular raptors and RNA viruses [59]. The eukaryal lineage diversified much later and possibly after the endosymbiosis of already diversified bacterial species. Our data is thus also incompatible with the hypothesis suggesting that eukaryotes originated from within Archaea (based on gene sequences) ([67]; see [59] for critique). In turn, the new ToL supported previously published analysis of similar kind where the use of conserved protein domain and RNA structures led to topologies favoring an ancient thermophilic origin of the archaeal superkingdom and the three-domain topology [15, 17].

More generally, our phylogenies are incompatible with previously published phylogenies from gene sequences that do not take into consideration the heterogeneous history of individual protein domains and their associated molecular functions. We argue that phylogenies built from gene sequences do not truly reflect the evolutionary history of entire organisms. We note that gene sequences are prone to high rates of mutations [68] and suffer from a number of phylogenetic artifacts including problems resulting from sequence alignments, insertions/deletions, and interactions of sequence sites to produce domain structures thus violating the assumption of character independence (discussed in detail elsewhere [13]). Thus, genes and their sequences cannot be considered conserved evolutionary units and do not make reliable markers for phylogenetic studies involving deep comparisons. In contrast, protein domain structures are more conserved than gene sequences and have been utilized in the past to reliably uncover very deep evolutionary relationships among superkingdoms [15–18]. Our phylogenies based on the genomic census of molecular functions are also compatible with the protein domain and RNA structure phylogenies and thus should be considered equally robust. Most importantly, GO terms approximate the physiology of an organism and truly depict a ToL (e.g., [29]). Furthermore, the use of terminal GO terms increases the resolution not only in the most basal branches of the ToL (a large number of ancient GO_TMF_ terms had very high *f* values thus providing an extended set of conserved characters) but also in the very derived (terminal terms represent highly specialized molecular functions that may not be conserved across all taxa). In light of these considerations, our finding that the root of cellular life is in thermophilic Archaea is a significant outcome that is supported by sound evolutionary and technical considerations.

### 3.6. Reliability of Our Study

In this study, we used GO terms that were both manually and electronically curated without reference to their evidential codes. We have shown previously that different evidence codes lead to similar tree topologies and, consequently, do not compromise our conclusions (e.g., [35]). Our study is also robust against the effects of the 50% GO coverage threshold as organisms with varying GO coverage (Table S1) were still well positioned in the ToL (Figure 3) and unequal sampling of taxa from superkingdoms [18]. However, it relies heavily on the current GO annotations and information about organism lifestyles. GO definitions are constantly updated and new relationships are introduced. Moreover, the fundamental assumption behind the evolutionary groupings of organisms and GO history is the existence of shared and derived patterns in the occurrence and abundance of their molecular functions, which complies with Weston's generality criterion of character polarization [69] and is hardly unreasonable. These factors should be taken into consideration when interpreting our conclusions. We expect however that phylogenomic patterns described in this study will remain robust with data growth and that significant revisions would be unlikely.

The phylogenetic characters used in this study are GO terms that provide ontological definitions linked to protein structures and gene products. Compared to other available molecular characters (e.g., gene sequences), these definitions better approximate the physiology of organisms and provide the ideal set of characters to make systemic evolutionary statements at organism level. Molecular functions are also refractory to problems of evolutionary reticulation; they are tightly linked to domain organization in proteins [49], which is minimally affected by convergent evolution [70]. We have previously used GO terms to study their evolutionary impact on cells (e.g., [28, 29]). The novelty here was to dissect their origin and spread in major superkingdoms. This exercise provides strong support to previously conducted comparative functionomic analysis [28] and a ToL reconstructed from the census of GO terms [29].

We note that forces that govern the evolution of genes and proteins are also applicable to the evolution of molecular functions. For example, gene duplication followed by neofunctionalization can create functional novelty [71]. Recently, Bacteria were shown to “rewire” metabolic networks by loss (rather than gain!) of functions when faced with environmental stress [72]. This implies that both gain and loss of functions may be important in akaryotes. Another useful contribution to the functional repertoire comes from HGT [73]. This was demonstrated with an example of transfer of “penicillin binding” molecular activity from Bacteria to Archaea. Finally,* de novo* gene creation cannot be underappreciated. Novel genes enhance the functional capabilities in cells, especially in eukaryotes. Another selective pressure that may trigger the appearance of new functions is the pressure of pathogens, especially RNA viruses. RNA viruses mutate at much higher rates and immune systems in higher-order eukaryotes are thus likely to acquire new molecular activities to combat invading pathogens.

To our knowledge, our new methodology supports the application of gene ontologies in evolutionary studies and is an innovative addition to the toolkit of molecular characters used in phylogenetic analysis. However, and on a balanced note, GO terms are prone to homoplasious events such as convergent evolution and HGT that arise from functional recruitment. To protect from these effects, we carefully excluded 115 GO terms that were identified as probable candidates of HGT. Furthermore, we restricted the analysis to include only terminal GO terms as they represent the most specialized functional annotations and thus may not be conserved across all taxa. We also excluded parasitic and facultative parasitic organisms from the analysis as they coevolve with their hosts and complicate phylogenetic analysis. During all these steps, we minimized the number of characters that were probable candidates suffering from homoplasy. The final dataset of 249 free-living genomes and a repertoire of 1,924 GO_TMF_ terms should be considered minimally affected by homoplasious events from HGT, parallel and convergent evolution, and biases introduced by differences in organism lifestyles. Indeed, phylogenies based on the genomic census of molecular functions performed in a way superior to the ToL reconstructed from rRNA gene trees in resolving phylogenetic relationships of major taxonomic groups of living organisms, at a cost of slightly decreasing rescaled consistency indices (RI) (from ~0.7 for ToLs reconstructed from protein domains to ~0.6 for ToLs reconstructed from GO terms) [29]. In these studies, the impact of nonvertical evolution on the phylogenies built from the genomic census of molecular functions is expected to be minimal since ToFs were congruent with trees of domains previously built from protein domain structures. ToL reconstruction is a difficult problem affected by a number of methodological and biological complications. However, our methodology should be considered equally (if not more) robust to the other existing approaches, a better approximation to the problem of phylogenetic analysis of species, and a new direction to the future use of GO terms in phylogenetic analyses that carries the potential of improvement.

## 4. Conclusions

Our investigations revealed that the roots of cellular life lie in the ancestors of thermophilic archaeal species. This is an interesting but atypical conclusion that is supported by both the distribution of molecular functions in the genomes of dozens of cellular species and the phylogenomic approach of ideographic analysis. The use of GO terms as molecular characters provided significant insights into the functioning and evolution of superkingdoms. In particular, we uncovered remarkable functional diversity of the eukaryotic genomes, which displayed a burst of appearance of novel molecular functions relatively late in evolution. In comparison, proteomes of akaryotic microbes persisted by favoring economy and following a minimalist path. The analysis puts forth the concept that GO definitions are useful and reliable characters for use in phylogenetic studies. Despite their complex hierarchical organization and vulnerability to the forces of recruitment, these new phylogenetic characters carry enormous potential to resolve phylogenies depicting natural history.

## Supplementary Material

List of free-living organisms sampled in this study along with taxonomic classification and GO coverage.

## Figures and Tables

**Table 1:** List of universal GO_TMF_ terms present in all 249 sampled genomes, sorted by *nd* values (ascending) (modified from [28]).

GO Id	GO Name	Age (*nd*)	Distribution Index (*f*)
GO:0005524	ATP binding	0	1
GO:0008270	zinc ion binding	0.005	1
GO:0000287	magnesium ion binding	0.009	1
GO:0005525	GTP binding	0.014	1
GO:0004222	metalloendopeptidase activity	0.023	1
GO:0010181	FMN binding	0.028	1
GO:0030145	manganese ion binding	0.033	1
GO:0003924	GTPase activity	0.038	1
GO:0003887	DNA-directed DNA polymerase activity	0.042	1
GO:0004252	serine-type endopeptidase activity	0.047	1
GO:0003746	translation elongation factor activity	0.052	1
GO:0009982	pseudouridine synthase activity	0.056	1
GO:0004523	ribonuclease H activity	0.103	1
GO:0004826	phenylalanine-tRNA ligase activity	0.108	1
GO:0004821	histidine-tRNA ligase activity	0.127	1
GO:0004820	glycine-tRNA ligase activity	0.127	1
GO:0004824	lysine-tRNA ligase activity	0.136	1
GO:0004831	tyrosine-tRNA ligase activity	0.150	1
GO:0004618	phosphoglycerate kinase activity	0.169	1
GO:0004634	phosphopyruvate hydratase activity	0.174	1
GO:0004749	ribose phosphate diphosphokinase activity	0.174	1
GO:0003952	NAD+ synthase (glutamine-hydrolyzing) activity	0.178	1
GO:0004815	aspartate-tRNA ligase activity	0.183	1
GO:0004807	triose-phosphate isomerase activity	0.183	1
GO:0004813	alanine-tRNA ligase activity	0.188	1
GO:0003917	DNA topoisomerase type I activity	0.192	1

**Table 2:** List of outlier GO_TMF_ terms in superkingdom taxonomic groups.

Taxonomic group	GO Id	GO Name	Age (*nd*)	Distribution Index (*f*)
ABE	GO:0003810	protein-glutamine gamma-glutamyltransferase activity	0.97	0.06
ABE	GO:0004715	non-membrane spanning protein tyrosine kinase activity	1	0.18

AB	GO:0008658	penicillin binding	0.08	0.76
AB	GO:0015415	phosphate ion transmembrane-transporting atpase activity	0.21	0.85
AB	GO:0009030	thiamine-phosphate kinase activity	0.21	0.69
AB	GO:0008966	phosphoglucosamine mutase activity	0.22	0.76
AB	GO:0015412	molybdate transmembrane-transporting atpase activity	0.22	0.66
AB	GO:0019134	glucosamine-1-phosphate N-acetyltransferase activity	0.23	0.66
AB	GO:0008881	glutamate racemase activity	0.23	0.65
AB	GO:0008763	UDP-N-acetylmuramate-L-alanine ligase activity	0.24	0.73
AB	GO:0008784	alanine racemase activity	0.24	0.73
AB	GO:0008760	UDP-N-acetylglucosamine 1-carboxyvinyltransferase activity	0.25	0.61
AB	GO:0008965	phosphoenolpyruvate-protein phosphotransferase activity	0.25	0.57
AB	GO:0008984	protein-glutamate methylesterase activity	0.25	0.59
AB	GO:0000286	alanine dehydrogenase activity	0.27	0.48
AB	GO:0016960	ribonucleoside-diphosphate reductase activity, thioredoxin disulfide as acceptor	0.28	0.53
AB	GO:0008855	exodeoxyribonuclease VII activity	0.31	0.72
AB	GO:0009381	excinuclease ABC activity	0.31	0.80

B	GO:0008766	UDP-N-acetylmuramoylalanyl-D-glutamyl-2,6-diaminopimelate-D-alanyl-D-alanine ligase activity	0.24	0.73
B	GO:0008961	phosphatidylglycerol-prolipoprotein diacylglyceryl transferase activity	0.25	0.64
B	GO:0008832	dGTPase activity	0.26	0.55
B	GO:0009002	serine-type D-Ala-D-Ala carboxypeptidase activity	0.31	0.60
B	GO:0008882	[glutamate-ammonia-ligase] adenylyltransferase activity	0.36	0.41
B	GO:0008914	leucyltransferase activity	0.36	0.45
B	GO:0019146	arabinose-5-phosphate isomerase activity	0.38	0.31
B	GO:0019143	3-deoxy-manno-octulosonate-8-phosphatase activity	0.38	0.33
B	GO:0004456	phosphogluconate dehydratase activity	0.38	0.23
B	GO:0008693	3-hydroxydecanoyl-[acyl-carrier-protein] dehydratase activity	0.38	0.22
B	GO:0008918	lipopolysaccharide 3-alpha-galactosyltransferase activity	0.66	0.01
B	GO:0030733	fatty acid O-methyltransferase activity	0.66	0.00

AE	GO:0004579	dolichyl-diphosphooligosaccharide-protein glycotransferase activity	0.77	0.10
AE	GO:0004965	G-protein coupled GABA receptor activity	0.93	0.05
