# Supplementary material for: A Phylogenomic Census of Molecular Functions Identifies Modern Thermophilic Archaea as the Most Ancient Form of Cellular Life
Source: Archaea. 2014 Aug 31;2014:706468. doi: 10.1155/2014/706468 (PMC4164138; doi:10.1155/2014/706468)
Supplement: Supplementary file 1 — List of free-living organisms sampled in this study along with taxonomic classification and GO coverage. [file 706468.f1.pdf]

SUPPLEMENTARY TABLE 1. Scientific names of free-living organisms sampled in this study along with taxonomic classification and GO coverage.

#	Organism	Taxonomy	Superkingdom	GO coverage (%)
1	*Aeropyrum pernix K1*	Crenarchaeota-Desulfurococcales	Archaea	56
2	*Sulfolobus solfataricus P2*	Crenarchaeota- Sulfolobales	Archaea	52
3	*Sulfolobus acidocaldarius DSM 639*	Crenarchaeota-Sulfolobales	Archaea	54
4	*Thermofilum pendens Hrk 5*	Crenarchaeota-Thermoproteales	Archaea	53
5	*Pyrobaculum islandicum DSM 4184*	Crenarchaeota- Thermoproteales	Archaea	52
6	*Caldivirga maquilingensis IC-167*	Crenarchaeota- Thermoproteales	Archaea	58
7	*Metallosphaera sedula DSM 5348*	Crenarchaeota- Sulfolobales	Archaea	55
8	*Staphylothermus marinus F1*	Crenarchaeota- Desulfurococcales	Archaea	55
9	*Pyrobaculum calidifontis JCM 11548*	Crenarchaeota- Thermoproteales	Archaea	52
10	*Hyperthermus butylicus DSM 5456*	Crenarchaeota- Desulfurococcales	Archaea	54
11	*Sulfolobus islandicus L.S.2.15*	Crenarchaeota- Sulfolobales	Archaea	54
12	*Thermoproteus neutrophilus V24Sta*	Crenarchaeota- Thermoproteales	Archaea	51
13	*Desulfurococcus kamchatkensis 1221n*	Crenarchaeota- Desulfurococcales	Archaea	55
14	*Methanosphaerula palustris E1-9c*	Euryarchaeota- Methanomicrobia	Archaea	57
15	*Archaeoglobus fulgidus DSM 4304*	Euryarchaeota-Archaeoglobi	Archaea	56
16	*Natronomonas pharaonis DSM 2160*	Euryarchaeota-Halobacteria	Archaea	52
17	*Haloquadratum walsbyi DSM 16790*	Euryarchaeota-Halobacteria	Archaea	51
18	*Halobacterium salinarum R1*	Euryarchaeota-Halobacteria	Archaea	51
19	*Methanobacterium thermoautotrophicum*	Euryarchaeota-Methanobacteria	Archaea	58
20	*Methanosphaera stadtmanae DSM 3091*	Euryarchaeota-Methanobacteria	Archaea	61
21	*Methanobrevibacter smithii PS*	Euryarchaeota-Methanobacteria	Archaea	59
22	*Methanocaldococcus jannaschii DSM 2661*	Euryarchaeota-Methanococci	Archaea	58
23	*Methanococcus vannielii SB*	Euryarchaeota-Methanococci	Archaea	63
24	*Methanococcus aeolicus Nankai-3*	Euryarchaeota-Methanococci	Archaea	65
25	*Methanococcus maripaludis C7*	Euryarchaeota-Methanococci	Archaea	61
26	*Methanosarcina mazei Go1*	Euryarchaeota-Methanomicrobia	Archaea	53
27	*Methanococcoides burtonii DSM 6242*	Euryarchaeota-Methanomicrobia	Archaea	58
28	*Methanosarcina barkeri*	Euryarchaeota-Methanomicrobia	Archaea	52
29	*Methanospirillum hungatei JF-1*	Euryarchaeota-Methanomicrobia	Archaea	57
30	*Methanosaeta thermophila PT*	Euryarchaeota-Methanomicrobia	Archaea	62
31	*Methanoculleus marisnigri JR1*	Euryarchaeota-Methanomicrobia	Archaea	56
32	*Methanocorpusculum labreanum Z*	Euryarchaeota-Methanomicrobia	Archaea	61
33	*Methanoregula boonei 6A8*	Euryarchaeota-Methanomicrobia	Archaea	58
34	*Methanopyrus kandleri AV19*	Euryarchaeota-Methanopyri	Archaea	56
35	*Pyrococcus kodakaraensis KOD1*	Euryarchaeota-Thermococci	Archaea	52
36	*Pyrococcus furiosus*	Euryarchaeota-Thermococci	Archaea	58
37	*Pyrococcus abyssi Orsay*	Euryarchaeota-Thermococci	Archaea	63
38	*Thermococcus onnurineus NA1*	Euryarchaeota-Thermococci	Archaea	59

39	*Thermococcus gammatolerans EJ3*	Euryarchaeota-Thermococci	Archaea	56
40	*Thermococcus sibiricus MM 739*	Euryarchaeota-Thermococci	Archaea	57
41	*Picrophilus torridus DSM 9790*	Euryarchaeota-Thermoplasmata	Archaea	66
42	*Thermoplasma acidophilum DSM 1728*	Euryarchaeota-Thermoplasmata	Archaea	61
43	*Thermoplasma volcanium GSS1*	Euryarchaeota-Thermoplasmata	Archaea	60
44	*Korarchaeum cryptofilum OPF8*	Korarchaeota	Archaea	61
45	*Nitrosopumilus maritimus SCM1*	Thaumarchaeota	Archaea	53
46	*Acidobacteria bacterium*	Acidobacteria	Bacteria	54
47	*Solibacter usitatus Ellin6076*	Acidobacteria	Bacteria	54
48	*Acidobacterium capsulatum ATCC 51196*	Acidobacteria	Bacteria	57
49	*Nocardioides sp.*	Actinobacteria	Bacteria	60
50	*Corynebacterium efficiens TS-314*	Actinobacteria	Bacteria	56
51	*Streptomyces avermitilis MA-4680*	Actinobacteria	Bacteria	57
52	*Rhodococcus erythropolis PR4*	Actinobacteria	Bacteria	59
53	*Mycobacterium avium 104*	Actinobacteria	Bacteria	59
54	*Nocardia farcinica IFM 10152*	Actinobacteria	Bacteria	54
55	*Rubrobacter xylanophilus DSM 9941*	Actinobacteria	Bacteria	68
56	*Kineococcus radiotolerans SRS30216*	Actinobacteria	Bacteria	57
57	*Thermobifida fusca YX*	Actinobacteria	Bacteria	60
58	*Arthrobacter aurescens TC1*	Actinobacteria	Bacteria	61
59	*Acidothermus cellulolyticus 11B*	Actinobacteria	Bacteria	65
60	*Kocuria rhizophila*	Actinobacteria	Bacteria	65
61	*Salinispora arenicola CNS-205*	Actinobacteria	Bacteria	58
62	*Saccharopolyspora erythraea NRRL 2338*	Actinobacteria	Bacteria	61
63	*Micrococcus luteus NCTC 2665*	Actinobacteria	Bacteria	64
64	*Beutenbergia cavernae DSM 12333*	Actinobacteria	Bacteria	64
65	*Gordonia bronchialis DSM 43247*	Actinobacteria	Bacteria	55
66	*Persephonella marina L2TR*	Aquificae	Bacteria	59
67	*Sulfurihydrogenibium azorense Az-Fu1*	Aquificae	Bacteria	64
68	*Aquifex aeolicus VF5*	Aquificae	Bacteria	67
69	*Cytophaga hutchinsonii ATCC 33406*	Bacteroidetes	Bacteria	50
70	*Salinibacter ruber DSM 13855*	Bacteroidetes	Bacteria	59
71	*Flavobacterium johnsoniae*	Bacteroidetes	Bacteria	52
72	*Gramella forsetii KT0803*	Bacteroidetes	Bacteria	52
73	*Pedobacter heparinus DSM 2366*	Bacteroidetes	Bacteria	52
74	*Flavobacteriaceae bacterium 3519-10*	Bacteroidetes	Bacteria	50
75	*Chlorobium limicola DSM 245*	Chlorobi	Bacteria	60
76	*Prosthecochloris aestuarii DSM 271*	Chlorobi	Bacteria	61
77	*Pelodictyon luteolum DSM 273*	Chlorobi	Bacteria	65
78	*Chlorobaculum parvum thiosulfatophilum*	Chlorobi	Bacteria	64
79	*Chloroherpeton thalassium*	Chlorobi	Bacteria	60
80	*Thermomicrobium roseum DSM 5159*	Chloroflexi	Bacteria	56

81	*Herpetosiphon aurantiacus ATCC 23779*	Chloroflexi	Bacteria	55
82	*Chloroflexus aurantiacus ATCC 29366*	Chloroflexi	Bacteria	62
83	*Roseiflexus castenholzii DSM 13941*	Chloroflexi	Bacteria	59
84	*Synechococcus elongatus PCC 7942*	Cyanobacteria	Bacteria	57
85	*Synechocystis sp.*	Cyanobacteria	Bacteria	52
86	*Prochlorococcus marinus MIT 9211*	Cyanobacteria	Bacteria	57
87	*Trichodesmium erythraeum ISM101*	Cyanobacteria	Bacteria	51
88	*Anabaena variabilis ATCC 29413*	Cyanobacteria	Bacteria	52
89	*Cyanothece sp. PCC 7425*	Cyanobacteria	Bacteria	50
90	*Deinococcus radiodurans R1*	Deinococcus-Thermus	Bacteria	54
91	*Thermus aquaticus Y51MC23*	Deinococcus-Thermus	Bacteria	56
92	*Dictyoglomus thermophilum H-6-12*	Dictyoglomi	Bacteria	66
93	*Leuconostoc mesenteroides ATCC 8293*	Firmicutes	Bacteria	65
94	*Oenococcus oeni PSU-1*	Firmicutes	Bacteria	66
95	*Oceanobacillus iheyensis HTE831*	Firmicutes	Bacteria	61
96	*Bacillus subtilis*	Firmicutes	Bacteria	60
97	*Geobacillus kaustophilus HTA426*	Firmicutes	Bacteria	57
98	*Carboxydothermus hydrogenoformans Z-2901*	Firmicutes	Bacteria	57
99	*Exiguobacterium sibiricum 255-15*	Firmicutes	Bacteria	61
100	*Streptococcus thermophilus ATCC BAA-250*	Firmicutes	Bacteria	63
101	*Moorella thermoacetica ATCC 39073*	Firmicutes	Bacteria	63
102	*Clostridium acetobutylicum ATCC 824*	Firmicutes	Bacteria	59
103	*Desulfitobacterium hafniense DCB-2*	Firmicutes	Bacteria	58
104	*Lactococcus lactis SK11*	Firmicutes	Bacteria	61
105	*Listeria innocua Clip11262*	Firmicutes	Bacteria	60
106	*Pediococcus pentosaceus ATCC 25745*	Firmicutes	Bacteria	65
107	*Alkaliphilus metalliredigens QYMF*	Firmicutes	Bacteria	56
108	*Coprothermobacter proteolyticus DSM 5265*	Firmicutes	Bacteria	64
109	*Syntrophomonas wolfei*	Firmicutes	Bacteria	59
110	*Thermoanaerobacter pseudethanolicus ATCC 33223*	Firmicutes	Bacteria	65
111	*Desulfotomaculum reducens MI-1*	Firmicutes	Bacteria	60
112	*Caldicellulosiruptor saccharolyticus DSM 8903*	Firmicutes	Bacteria	61
113	*Brevibacillus brevis NBRC 100599*	Firmicutes	Bacteria	55
114	*Halothermothrix orenii H 168*	Firmicutes	Bacteria	65
115	*Lactobacillus delbrueckii ATCC 11842*	Firmicutes	Bacteria	62
116	*Staphylococcus carnosus*	Firmicutes	Bacteria	64
117	*Natranaerobius thermophilus JW/NM-WN-LF*	Firmicutes	Bacteria	60
118	*Macrococcus caseolyticus JCSC5402*	Firmicutes	Bacteria	64
119	*Blautia hydrogenotrophica DSM 10507*	Firmicutes	Bacteria	52
120	*Desulforudis audaxviator MP104C*	Firmicutes	Bacteria	60
121	*Anoxybacillus flavithermus WK1*	Firmicutes	Bacteria	61

122	*Heliobacterium modesticaldum Ice1*	Firmicutes	Bacteria	54
123	*Eubacterium biforme DSM 3989*	Firmicutes	Bacteria	51
124	*Anaerocellum thermophilum DSM 6725*	Firmicutes	Bacteria	56
125	*Anaerococcus hydrogenalis DSM 7454*	Firmicutes	Bacteria	57
126	*Gemmatimonas aurantiaca*	Gemmatimonadetes	Bacteria	55
127	*Thermodesulfovibrio yellowstonii DSM 11347*	Nitrospirae	Bacteria	65
128	*Xanthobacter autotrophicus Py2*	Proteobacteria-Alpha	Bacteria	60
129	*Hyphomonas neptunium ATCC 15444*	Proteobacteria-Alpha	Bacteria	60
130	*Silicibacter pomeroyi DSS-3*	Proteobacteria-Alpha	Bacteria	63
131	*Rhodopseudomonas palustris ATCC BAA-98*	Proteobacteria-Alpha	Bacteria	62
132	*Zymomonas mobilis*	Proteobacteria-Alpha	Bacteria	58
133	*Rhodospirillum rubrum ATCC 11170*	Proteobacteria-Alpha	Bacteria	64
134	*Novosphingobium aromaticivorans DSM 12444*	Proteobacteria-Alpha	Bacteria	62
135	*Jannaschia sp.*	Proteobacteria-Alpha	Bacteria	58
136	*Gluconobacter oxydans 621H*	Proteobacteria-Alpha	Bacteria	60
137	*Erythrobacter litoralis HTCC2584*	Proteobacteria-Alpha	Bacteria	55
138	*Sphingopyxis alaskensis RB2256*	Proteobacteria-Alpha	Bacteria	61
139	*Paracoccus denitrificans PD1222*	Proteobacteria-Alpha	Bacteria	61
140	*Nitrobacter winogradskyi Nb-255*	Proteobacteria-Alpha	Bacteria	56
141	*Pelagibacter ubique HTCC1062*	Proteobacteria-Alpha	Bacteria	71
142	*Rhodoferax ferrireducens T118*	Proteobacteria-Alpha	Bacteria	63
143	*Magnetospirillum magneticum AMB-1*	Proteobacteria-Alpha	Bacteria	56
144	*Rhodobacter sphaeroides ATCC 17029*	Proteobacteria-Alpha	Bacteria	62
145	*Acidiphilium cryptum JF-5*	Proteobacteria-Alpha	Bacteria	65
146	*Roseobacter denitrificans OCh 114*	Proteobacteria-Alpha	Bacteria	59
147	*Granulibacter bethesdensis CGDNIH1*	Proteobacteria-Alpha	Bacteria	66
148	*Sphingomonas wittichii RW1*	Proteobacteria-Alpha	Bacteria	65
149	*Maricaulis maris MCS10*	Proteobacteria-Alpha	Bacteria	59
150	*Beijerinckia indica*	Proteobacteria-Alpha	Bacteria	60
151	*Methylocella silvestris BL2*	Proteobacteria-Alpha	Bacteria	58
152	*Dinoroseobacter shibae DFL 12*	Proteobacteria-Alpha	Bacteria	60
153	*Parvibaculum lavamentivorans DS-1*	Proteobacteria-Alpha	Bacteria	62
154	*Ochrobactrum anthropi ATCC 49188*	Proteobacteria-Alpha	Bacteria	62
155	*Methylobacterium chloromethanicum CM4*	Proteobacteria-Alpha	Bacteria	53
156	*Oligotropha carboxidovorans OM5*	Proteobacteria-Alpha	Bacteria	53
157	*Caulobacter crescentus NA1000*	Proteobacteria-Alpha	Bacteria	61
158	*Hirschia baltica ATCC 49814*	Proteobacteria-Alpha	Bacteria	57
159	*Dechloromonas aromatica RCB*	Proteobacteria-Beta	Bacteria	60
160	*Herminiimonas arsenicoxydans*	Proteobacteria-Beta	Bacteria	59
161	*Burkholderia cepacia J2315*	Proteobacteria-Beta	Bacteria	62
162	*Nitrosomonas europaea ATCC 19718*	Proteobacteria-Beta	Bacteria	62
163	*Acidovorax sp.*	Proteobacteria-Beta	Bacteria	59

164	*Chromobacterium violaceum ATCC 12472*	Proteobacteria-Beta	Bacteria	58
165	*Methylobacillus flagellatus KT*	Proteobacteria-Beta	Bacteria	63
166	*Thiobacillus denitrificans ATCC 25259*	Proteobacteria-Beta	Bacteria	60
167	*Polynucleobacter sp.*	Proteobacteria-Beta	Bacteria	65
168	*Nitrosospira multiformis ATCC 25196*	Proteobacteria-Beta	Bacteria	60
169	*Polaromonas naphthalenivorans CJ2*	Proteobacteria-Beta	Bacteria	56
170	*Janthinobacterium sp.*	Proteobacteria-Beta	Bacteria	61
171	*Ralstonia eutropha ATCC 17699*	Proteobacteria-Beta	Bacteria	66
172	*Leptothrix cholodnii SP-6*	Proteobacteria-Beta	Bacteria	63
173	*Delftia acidovorans SPH-1*	Proteobacteria-Beta	Bacteria	59
174	*Methylibium petroleiphilum PM1*	Proteobacteria-Beta	Bacteria	57
175	*Variovorax paradoxus S110*	Proteobacteria-Beta	Bacteria	62
176	*Methylotenera mobilis JLW8*	Proteobacteria-Beta	Bacteria	62
177	*Syntrophus aciditrophicus SB*	Proteobacteria-Delta	Bacteria	55
178	*Desulfococcus oleovorans Hxd3*	Proteobacteria-Delta	Bacteria	59
179	*Desulfobacterium autotrophicum HRM2*	Proteobacteria-Delta	Bacteria	61
180	*Desulfotalea psychrophila LSv54*	Proteobacteria-Delta	Bacteria	54
181	*Geobacter metallireducens GS-15*	Proteobacteria-Delta	Bacteria	63
182	*Anaeromyxobacter dehalogenans 2CP-C*	Proteobacteria-Delta	Bacteria	59
183	*Syntrophobacter fumaroxidans MPOB*	Proteobacteria-Delta	Bacteria	58
184	*Pelobacter carbinolicus DSM 2380*	Proteobacteria-Delta	Bacteria	60
185	*Desulfatibacillum alkenivorans AK-01*	Proteobacteria-Delta	Bacteria	58
186	*Haliangium ochraceum DSM 14365*	Proteobacteria-Delta	Bacteria	50
187	*Desulfovibrio desulfuricans ATCC 27774*	Proteobacteria-Delta	Bacteria	63
188	*Sulfurimonas denitrificans ATCC 33889*	Proteobacteria-Epsilon	Bacteria	64
189	*Arcobacter butzleri RM4018*	Proteobacteria-Epsilon	Bacteria	62
190	*Nitratiruptor sp.*	Proteobacteria-Epsilon	Bacteria	65
191	*Sulfurovum sp.*	Proteobacteria-Epsilon	Bacteria	58
192	*Colwellia psychrerythraea 34H*	Proteobacteria-Gamma	Bacteria	53
193	*Alkalilimnicola ehrlichei MLHE-1*	Proteobacteria-Gamma	Bacteria	63
194	*Saccharophagus degradans ATCC 204508*	Proteobacteria-Gamma	Bacteria	57
195	*Pseudomonas aeruginosa LMG 12228*	Proteobacteria-Gamma	Bacteria	63
196	*Methylococcus capsulatus*	Proteobacteria-Gamma	Bacteria	60
197	*Psychrobacter arcticus 273-4*	Proteobacteria-Gamma	Bacteria	63
198	*Idiomarina loihiensis ATCC 15444*	Proteobacteria-Gamma	Bacteria	66
199	*Citrobacter koseri ATCC BAA-895*	Proteobacteria-Gamma	Bacteria	55
200	*Enterobacter sakazakii ATCC BAA-894*	Proteobacteria-Gamma	Bacteria	55
201	*Chromohalobacter salexigens DSM 3043*	Proteobacteria-Gamma	Bacteria	66
202	*Alteromonas macleodii*	Proteobacteria-Gamma	Bacteria	56
203	*Thiomicrospira crunogena XCL-2*	Proteobacteria-Gamma	Bacteria	64
204	*Shewanella putrefaciens CN-32*	Proteobacteria-Gamma	Bacteria	60

205	*Azotobacter vinelandii DJ*	Proteobacteria-Gamma	Bacteria	57
206	*Nitrosococcus oceani ATCC 19707*	Proteobacteria-Gamma	Bacteria	58
207	*Pseudoalteromonas haloplanktis TAC125*	Proteobacteria-Gamma	Bacteria	59
208	*Halorhodospira halophila SL1*	Proteobacteria-Gamma	Bacteria	66
209	*Hahella chejuensis KCTC 2396*	Proteobacteria-Gamma	Bacteria	50
210	*Marinobacter aquaeolei VT8*	Proteobacteria-Gamma	Bacteria	59
211	*Psychromonas ingrahamii 37*	Proteobacteria-Gamma	Bacteria	62
212	*Acidithiobacillus ferrooxidans ATCC 53993*	Proteobacteria-Gamma	Bacteria	59
213	*Aeromonas hydrophila*	Proteobacteria-Gamma	Bacteria	65
214	*Alcanivorax borkumensis SK2*	Proteobacteria-Gamma	Bacteria	64
215	*Marinomonas sp.*	Proteobacteria-Gamma	Bacteria	65
216	*Acinetobacter baumannii AB307-0294*	Proteobacteria-Gamma	Bacteria	59
217	*Vibrio cholerae MJ-1236*	Proteobacteria-Gamma	Bacteria	63
218	*Tolumonas auensis DSM 9187*	Proteobacteria-Gamma	Bacteria	66
219	*Leptospira biflexa Ames*	Spirochaetes	Bacteria	54
220	*Dethiosulfovibrio peptidovorans DSM 11002*	Synergistetes	Bacteria	62
221	*Thermanaerovibrio acidaminovorans DSM 6589*	Synergistetes	Bacteria	69
222	*Thermotoga maritima MSB8*	Thermotogae	Bacteria	65
223	*Fervidobacterium nodosum UW101*	Thermotogae	Bacteria	66
224	*Petrotoga mobilis SJ95*	Thermotogae	Bacteria	69
225	*Thermosipho africanus TCF52B*	Thermotogae	Bacteria	62
226	*Kosmotoga olearia TBF 19.5.1*	Thermotogae	Bacteria	65
227	*Opitutus terrae PB90-1*	Verrucomicrobia	Bacteria	56
228	*Methylacidiphilum infernorum V4*	Verrucomicrobia	Bacteria	52
229	*Aedes aegypti*	Arthropoda	Eukarya	56
230	*Drosophila melanogaster*	Arthropoda	Eukarya	65
231	*Anopheles gambiae*	Arthropoda	Eukarya	55
232	*Gallus gallus*	Chordata-Birds	Eukarya	78
233	*Danio rerio*	Chordata-Fish	Eukarya	64
234	*Bos taurus*	Chordata-Mammals	Eukarya	71
235	*Mus musculus*	Chordata-Mammals	Eukarya	67
236	*Rattus norvegicus*	Chordata-Mammals	Eukarya	78
237	*Homo sapiens*	Chordata-Primates	Eukarya	62
238	*Saccharomyces cerevisiae ATCC 204508*	Fungi-Ascomycota	Eukarya	82
239	*Emericella nidulans*	Fungi-Ascomycota	Eukarya	53
240	*Kluyveromyces lactis*	Fungi-Ascomycota	Eukarya	54
241	*Yarrowia lipolytica*	Fungi-Ascomycota	Eukarya	51
242	*Debaryomyces hansenii*	Fungi-Ascomycota	Eukarya	51
243	*Candida glabrata*	Fungi-Ascomycota	Eukarya	53
244	*Pichia stipitis*	Fungi-Ascomycota	Eukarya	55
245	*Aspergillus fumigatus FGSC A1100*	Fungi-Ascomycota	Eukarya	54
246	*Vanderwaltozyma polyspora DSM*	Fungi-Ascomycota	Eukarya	52

	70294			
247	*Cryptococcus neoformans JEC21*	Fungi-Basidiomycota	Eukarya	52
248	*Arabidopsis thaliana*	Plants-Streptophyta	Eukarya	51
249	*Vitis vinifera*	Plants-Streptophyta	Eukarya	53
